# Exploring the dynamic biopsychosocial health needs of middle-aged adults living alone: a qualitative study

**DOI:** 10.1080/17482631.2026.2632367

**Published:** 2026-02-20

**Authors:** Jaehee Yoon, En Jung Bae, Donghee Cho, Joohyun Chung, Heesook Son

**Affiliations:** a Seoul Wolchon Elementary School, Seoul, Republic of Korea; b Department of Nursing, Ansan University, Ansan-si, Gyeonggi-do, Republic of Korea; c Department of Nursing, Semyung University, Jecheon-si, Chungcheongbuk-do, Republic of Korea; d College of Nursing, University of Massachusetts, Amherst, MA, USA; e Red Cross College of Nursing, Chung-Ang University, Seoul, Republic of Korea

**Keywords:** Middle-aged adults, living alone, qualitative, health needs, biopsychosocial

## Abstract

**Introduction:**

The number of middle-aged adults living alone in South Korea is increasing, raising concerns about their physical, mental, and social health. This study explored their health concerns and perceived support needs.

**Methods:**

Semi-structured interviews were conducted with 12 adults aged 40–64 years living alone in urban and suburban areas. Purposive sampling ensured diverse experiences. Data were analyzed using deductive qualitative content analysis guided by the social-ecological model.

**Results:**

Five themes emerged: (1) navigating aging with autonomy and avoidance, (2) seeking companionship for a healthier solitary life, (3) pursuing personal growth through community engagement, (4) recognizing the influence of residential environments, and (5) managing health through social safety nets. Participants valued independence but experienced disconnection from traditional support systems. They emphasized the importance of social relationships, supportive living environments, and the absence of caregivers as a key concern.

**Conclusion:**

Tailored multilevel strategies are needed to support healthy aging among middle-aged adults living alone, including strengthening social connections, addressing caregiving gaps, and improving community and residential support.

## 1 Introduction

Aging populations, along with declining marriage rates and rising divorce rates, have contributed to a global increase in single-person households (Jacob et al., 2019; Song et al., 2018). From 2000 to 2023, the proportion of single-person households in the total population increased in many countries. Notably, the increase was more pronounced in South Korea than in the United States. It rose from 25.8% to 29.0% in the US (United States Census Bureau, 2023), whereas it increased from 15.5% to 34.5% in South Korea (Statistics Korea, 2024). Klinenberg (2016) emphasised that the global rise in solo living constitutes one of the most significant social changes of the modern era. This trend is especially pronounced among middle-aged adults (Statistics Korea, 2024) and is driven by delayed marriage, divorce, and shifting cultural norms (Song et al., 2018). Traditionally supported by family structures, this group now faces unique challenges that require revised health and social policies. Their health vulnerabilities represent an urgent issue that must be addressed at health and social policy levels (Raymo, 2015). However, their distinct health risks remain under-examined, both globally and in South Korea (Song et al., 2018). For instance, Chai and Mei (2022) reported that older Canadian individuals living alone were more likely to experience food insecurity, unhealthy lifestyles, and poorer self-rated health than their counterparts living with others.

Family dynamics significantly affect health (González-Rodríguez et al., 2024; Yeung et al., 2016), and living alone is associated with negative outcomes, including higher mortality (Zhao et al., 2022), psychological distress (Mazzuco et al., 2017; Stahl et al., 2017), unhealthy behaviours (Kim et al., 2017), and chronic diseases (Kwon et al., 2018; Park et al., 2014). Importantly, these health consequences are not confined to a single dimension but unfold across multiple layers of sociocultural context. Park and Choi (2015) demonstrate the intersection between economic insecurity and health inequality among middle-aged South Koreans living alone. Complementary qualitative studies further indicate that middle-aged adults perceive healthy aging as requiring not only physical and psychological well-being, but also financial stability and social support; being alone is often considered a major health risk in later life (Solhi et al., 2020; Solhi et al., 2022b). These findings underscore the importance of cultural and economic contexts alongside psychosocial factors in shaping health vulnerabilities.

Middle age marks changes in both roles and health statuses of individuals (Infurna et al., 2020). Conditions such as osteoporosis, cardiovascular disease, cognitive decline, and depression often arise or worsen (Harlow & Derby, 2015). Beyond physical changes, midlife is also a critical window for mental health promotion. In South Korea, suicide remains a major public health concern: the suicide rate was 26.2 per 100,000 in 2024 (OECD average: 10.8). Suicide accounted for 26.0% of deaths among adults aged 40–49 and was the second leading cause of death among those aged 50–59 (12.2%) (Ministry of Data and Statistics, 2024). In addition, the prevalence of depressive disorders increases from midlife into older age (e.g. 1.5% in ages 40–49, 1.8% in 50–59, and 3.1% in 70–79) (Statistics Korea, 2025). Therefore, middle age is a crucial period for preventing chronic illnesses and ensuring successful aging (Harlow & Derby, 2015; Rodrigues et al., 2023). Targeted interventions are urgently required (Infurna et al., 2020), especially as more middle-aged adults live alone and have increased health vulnerabilities.

The biopsychosocial (BPS) framework provides a comprehensive perspective for understanding the vulnerabilities of adults living alone. First introduced by Engel (1977), the model emphasises that health is shaped not only by biological, but also by psychological and social factors. Although widely applied in research, clinical practice, and guideline development, the model’s conceptual clarity and practical applicability have been debated. For instance, Wade and Halligan (2017) observed that, despite its use in complex interventions and its recognition by the World Health Organisation, the BPS framework has had limited influence on the broader organisation and funding of healthcare. These discussions highlight both its strengths and the continuing need to articulate its relevance in contemporary health contexts. Prior studies (Park & Choi, 2015; Solhi et al., 2020; Solhi et al., 2022b) have further demonstrated that the health of people living alone is influenced by interrelated biological, psychological, and social dimensions. Therefore, the BPS framework is particularly useful in this context as it integrates these factors into a single conceptual approach, enabling a more holistic understanding of health risks. It also provides a valuable basis for developing health policies and programs (Lehman et al., 2017; McLeroy et al., 1988). During middle age, individuals face distinct developmental tasks, including managing biological changes and chronic conditions, sustaining psychological resilience, and maintaining social relationships. These challenges underscore the appropriateness of applying the BPS framework to capture the unique health needs of middle-aged adults living alone in South Korea.

Qualitative research is vital for capturing lived experiences in context. Although some studies have explored health in middle-aged adults (Enjezab et al., 2012; Ghaljaei et al., 2017; Sharifi et al., 2014; Sharma & Suhail, 2022; Solhi et al., 2020; Solhi et al., 2022a; Zhang et al., 2024) or older adults living alone (Charpentier & Kirouac, 2022; Soulières & Charpentier, 2022), few have focused on the health needs of middle-aged individuals living alone. Preliminary research on unmarried middle-aged women has been limited in scope by gender (Kang & Seol, 2012). Moreover, few studies have examined how living conditions affect the overall well-being of single-person households in midlife.

To address these gaps, this study utilised the BPS framework to examine the multidimensional health needs of middle-aged South Koreans living alone. Through qualitative enquiry, we illuminated their lived experiences, generating evidence that extends beyond mere descriptive accounts to inform the design of tailored interventions. The findings of this study offer a solid foundation for developing targeted health strategies and policies in South Korea.

## 2 Methods

### 2.1 Study design

We conducted qualitative content analysis to examine the characteristics of middle-aged adults living alone, as well as their support systems.

### 2.2 Participants

Participants were selected using purposive sampling. Participants were recruited through community networks and online postings, and eligibility was confirmed based on predefined inclusion criteria. We defined middle-aged adults as individuals aged 40–64 years, consistent with South Korean demographic standards. “Living alone” refers to individuals residing without other adults or family members (Yeung & Cheung, 2015). To ensure sample homogeneity, participants were expected to have lived alone for over a year and never been married. Individuals who were divorced or widowed were excluded because their prior marital experiences and family histories were expected to shape distinct health perceptions and support needs. Those who met the inclusion criteria provided written informed consent. Twelve participants were included in this study.

### 2.3 Data collection

In-depth interviews were conducted online via Zoom at the participants’ convenience with the help of a semi-structured interview guide. Interviews took place from August 1 to October 31, 2023, and lasted 50–90 min each. Before each interview, the researcher explained the purpose and process of the study and assured the participants that they would have sufficient time to respond. At the end of each interview, the researcher summarised the key points and invited participants to make corrections or additions. Prompts were used to refocus the discussion when conversations deviated or stalled. The principal interview questions were informed by the dynamic biopsychosocial model of health (Lehman et al., 2017) and the WHOQOL-BREF domains (WHOQOL Group, 1998).

Interviews continued until data saturation was reached. Saturation was achieved with 12 participants, because no new themes or insights emerged beyond this point. Guest et al. (2006) reported that saturation often occurs within the first 12 interviews in relatively homogeneous groups, supporting the adequacy of our sample size. This was assessed iteratively after each set of interviews to ensure that diverse perspectives were captured.

### 2.4 Data analysis

The deductive approach to qualitative content analysis proposed by Elo and Kyngäs (2008) was applied to examine support measures based on the collected data. The analysis was conducted in three stages: preparation, organisation, and reporting. In the preparation stage, interview questions were formulated using the holistic BPS framework to explore the characteristics of middle-aged individuals living alone (Table I).

**Table I.** Interview questions.

Categories	Item
Initial	May I enquire about when you started living alone? Is there a specific reason behind your choice to live alone?
Physical health	How do you perceive your current physical health status? Are there any positive or negative health habits that are related to your current health status? May I enquire about your usual health management practices? Are there any aspects of health management that you find challenging? Are there any factors that you believe would be beneficial for managing your health?
Psychological health	Could you please share your current emotional state? How do you typically manage negative emotions such as stress or sadness? Are there any challenges you encounter when trying to cultivate positive emotions in your daily life? Are there any factors that contribute to fostering positive emotions for you?
Social health	May I enquire about your relationship with family members with whom you do not live? How many people in your immediate surroundings are there to share your personal issues or everyday difficulties? Are there any challenges in establishing such connections?
Environmental	How do you perceive your current living environment? In your opinion, how much does the living environment impact your daily life? Are there aspects of your living environment that you believe need improvement? What environmental factors do you consider most crucial for living alone? What are your thoughts on the local environment where you currently reside? How much do you think the local environment influences your lifestyle? Are there aspects of the local environment that you think need improvement? What local factors do you consider most important for living alone?
Finalisation	Is there anything else you would like to share regarding your lifestyle and self-management?

Data analysis was conducted manually, without the use of specialised qualitative software. Transcripts were managed with a word processor, codes were organised and compared in Microsoft Excel spreadsheets, and analytic memos were written throughout the process to document researcher reflections. An analysis matrix was created using McLeroy et al. (1988) social-ecological model, which comprises five levels: intrapersonal, interpersonal, organisational, community, and public policy. Participant responses and researcher reflections were recorded and transcribed verbatim. Transcripts were reviewed iteratively to identify recurring patterns and meaningful statements. Codes were categorised into the five levels of the model and independently reviewed. Discrepancies were resolved through consensus. Subcategories emerged inductively through comparative analysis, allowing insights beyond the deductive framework. Debriefing sessions were held to reduce bias. Results were summarised in tables with participant quotes, and validity was ensured through participant and expert reviews, with feedback incorporated before final documentation.

### 2.5 Ethical considerations

The purpose and procedures of the study were explained to participants. Written informed consent was obtained from all participants before the study began. Participants were assured of their right to withdraw at any time, and their participation was voluntary. This study was approved by the Institutional Review Board of the Chung-Ang University, to which the principal investigator is affiliated (1041078-20230512-HR-136).

### 2.6 Qualitative rigour

The researcher maintained neutrality to prevent influencing participants' responses. Participant statements were transcribed verbatim to minimise data distortion or omissions. Each transcript was repeatedly reviewed by the research team, and the analysis involved iterative comparisons between the data and interpretations. To enhance reliability, some participants verified that the derived themes and quotes accurately reflected the research findings.

## 3 Results

Table II presents the participants’ general characteristics. Table III provides a detailed analysis of the results.

**Table II.** General participant characteristics (N = 12).

Categories	Frequency (%)	Mean (SD)
Gender Women Men	6 (50) 6 (50)	
Age		44.3 (2.5)
Height (cm)		166.2 (8.1)
Weight (kg)		66.8 (12.9)
Educational level High school College Postgraduate	1 (8.3) 7 (58.3) 4 (33.3)	
Employment status Employed	12 (100)	
Monthly income (10,000 won)		339.8 (103.2)
Total period of living alone (years)		13.7 (5.7)
Chronic disease Yes No	3 (25) 9 (75)	
Current smoker Yes No	2 (16.7) 10 (83.3)	
Alcohol use Yes No	7 (58.3) 5 (41.7)	

*Note*. The median age of participants was 44.5 years (range: 40–49); and the median duration of living alone was 13.8 years (range: 3.6–20.5).

**Table III.** Main themes and codes.

Categories	Subcategories	Code
Intrapersonal level **First theme:** The onset of aging with autonomy and avoidance	Experiences of changes due to the onset of aging	Aging is a mandatory assignment
		Numb and dry emotions
		Thinking about death and the meaning of life
	Life of idleness and freedom	Living a simplified life
		Not easy to change a lethargic lifestyle
		The freedom to choose one’s way of life
	Life of being alone in old age that I want to avoid	Avoidance and passive preparation for situations in old age
		Loneliness and anxiety due to the absence of a caretaker
Interpersonal level **Second theme:** Anticipating the arrival of a companion for a healthy life in seclusion	Voluntary isolation	Being alone to avoid interpersonal conflict and hurt
		Being comfortable when alone
		Living a solitary life and not wanting to be seen by others
	Life that I want to live with a companion for health resources	Needing a companion to share one’s life and feel supported
		A companion who contributes to a healthy lifestyle
	Difficulties forming relationships owing to passivity and realistic constraints	Lack of active efforts to find a companion
		Relationships that do not develop because of practical conditions
		Limitations on relationships that stem from different lifestyles than those of family-oriented peers
		Lack of social networks for independent midlife
Organisational level **Third theme:** A life that grows within community-based organisations	Daily routines shaped by organisational structures	Living close to one’s organisation
		An organisation is a place where interactions occur naturally
	Organisations fostering individual growth through structured support	Organisations that provide opportunities for vitality and self-reflection
		Positive self-perceptions through interactions within the organisations
Community level **Fourth theme:** Importance of residential environments to the health and life of middle-aged individuals	Discomfort from a general perception of lacking achievement in personal development	The perception that middle age is a time when one must provide for oneself
		Feeling uncomfortable with the expectation that you should naturally be married
		Prejudice and self-doubt associated with being viewed as immature
		Perception of single-person households’ living as objects of admiration
	Pursuit of a safe residential environment that allows for social interaction	Psychological stability provided by a secure living space
		Concerns about the safety of one’s living environment
		Cramped living spaces that make it difficult to interact with others
		Life in a well-equipped place with facilities that help alleviate loneliness
Public policy level **Fifth theme:** Healthcare utilisation and health management improvement through social safety nets	Life outside the social safety net	A lack of social support policies for middle-aged individuals living alone
		Feeling a sense of injustice in a life marginalised by social blind spots
	Barriers to single-person household healthcare access and policy needs to improve health literacy	Difficulty in accessing healthcare without a caretaker
		Have not learned the skills to live alone
		Need to build an accessible platform for health information
		Need for a shared house where people can gain know-how and rely on each other

### 3.1 Intrapersonal level: Onset of aging with autonomy and avoidance

The intrapersonal level included three subcategories: “experiences of change due to aging,” “life of idleness and freedom,” and “life alone in old age to be avoided.” This theme explored participants’ perceptions of aging, including declining physical function, emotional fluctuations, and feelings of detachment. Participants preferred comfortable lives with minimal effort, and often prioritised current convenience over future health or financial planning. Despite vague anxieties, they were passive about preparing for aging and expressed concerns regarding future loneliness and a lack of caregivers. Examples include:

Subcategory 1: Experiences of change due to the onset of aging

*“In the past, my mood swings were severe; now, I have this comfortable emotional state... It's like, well, it can't be helped, so I just go with it or try to be happy...(but) I do want to enjoy many happy things. I want to keep many of them. But it feels like I've become numb a lot.” (Participant 3, male).*

This emotional shift was accompanied by recognition of physical decline and the way it shaped social interactions:

*“My eyesight is getting worse, and when I meet friends, all we talk about is health. It wasn’t like that in the past when we met… Now it’s more like, ‘This hurts,’ ‘This food is supposed to be good for you,’ or ‘I went to this place for treatment.’ Conversations revolve a lot around such topics.” (Participant 9, female).*

Subcategory 2: Life of idleness and freedom

*“Freedom can just lead to laziness, and there can be some oppression in supporting a family... It seems to have its pros and cons.” (Participant 2, male).*

While some participants emphasised the ambivalence of freedom, others described the everyday pleasures and conveniences it affords:

*“If someone were here with me, I might be forced to eat more. But as it is, I can sleep whenever I want, wake up late, and without anyone interfering, I can eat and sleep exactly when I feel like it. For me, that still feels like an advantage, and I still enjoy it.” (Participant 8, female).*

Subcategory 3: Life of being alone in old age that I want to avoid

*“I used to think about who would arrange my funeral, but it's not there... If my current boyfriend weren't by my side at that time, I would feel so lonely and [life would feel] so difficult.” (Participant 4, female).*

Beyond concerns about practical matters such as the end of life, participants also expressed ambivalence, oscillating between avoidance and underlying anxiety:

*“If I think too much about that stage, it feels overwhelming, so I haven’t thought about it deeply… Sometimes I feel anxious, sometimes afraid, but for now I still see it as something far away.” (Participant 9, female)*

### 3.2 Interpersonal level: anticipating the arrival of a companion for a healthy life in seclusion

The interpersonal level included three subcategories: “voluntary isolation,” “desiring companionship for health,” and “difficulties in forming relationships due to passivity and practical constraints.” This theme highlighted participants’ preference for solitude, despite their longing for meaningful social connections. They were hindered by limited social opportunities and peer networks, and tended to remain passive rather than actively pursue relationships. Examples include:

Subcategory 1: Voluntary isolation

*“As time goes by, I sometimes find it a bit harder to engage with people. After choosing to spend time quietly, I’ve been staying away from such gatherings for quite a while.” (Participant 3, male).*

In addition to distancing themselves from social gatherings, some participants emphasised a deliberate effort to set personal boundaries, even within existing relationships:

*“As I get older, I feel more strongly that there should be boundaries even among friends. Especially with people I meet through hobbies, I try not to cross a certain line, and I also refrain from opening up too much myself.” (Participant 4, female).*

Subcategory 2: Life that I want to live with a companion for health reasons

*“It's better to have a family together than be alone. When we're together, we can take care of each other and such...I think it's very important to share something, divide it, take care of each other emotionally and such.” (Participant 1, male).*

Beyond emphasising mutual emotional support, others highlighted the concrete benefits of companionship in maintaining healthier daily routines:

*“When it comes to meals, I tend to just put something or even forget at times. But if someone were with me, I would take better care of meals because I would also be looking after them… And if I could meet someone who truly understands me and talk about many things together, it would really help lift my mood.” (Participant 9, female).*

Subcategory 3: Difficulties forming relationships owing to passivity and realistic constraints

*“Before I became 40, I went on a lot of dates. But now, I don’t want to make that effort anymore. I could get married, but I don’t feel like putting in the effort.” (Participant 8, female).*

This sense of passivity was echoed by others who described the challenges of forming relationships not as a matter of unwillingness but as constrained by life stage and circumstances:

*“I don’t think there are many people who are voluntarily single. At my age, it’s not so much that I feel I must get married, but rather that I would like to, yet I find it difficult.” (Participant 11, male).*

### 3.3 Organisational level: a life that grows within community-based organisations

The organisational level included two subcategories: “daily routines shaped by organisational structures” and “organisations fostering growth through structured support.” This theme examined how workplaces, religious groups, and clubs provided structure, influencing participants’ daily activities and personal development. Participants reported that organisational rules and schedules enhanced stability, facilitated resource access, and promoted social interaction. These structured environments were essential for supporting participants’ psychological and emotional health and were distinct from informal community settings.

Subcategory 1: Daily routines shaped by organisational structures

*“I never go places more than 30 minutes away from my home and workplace. I always stay close... And the church I used to attend is only a five-minute walk from here.” (Participant 10, female).*

Others described how the spatial demands of work and religious life constrained their choices, leading them to situate themselves strategically between these institutions while also prioritising long-standing relationships:

*“Because I attend church, if I live closer to my workplace, the church becomes too far; but if I live closer to the church, then the workplace is far. So now I live exactly in between. I also prefer to maintain relationships with people I have already known and to continue interacting with them.” (Participant 9, female).*

Subcategory 2: Organisations fostering individual growth through structured support

*“Even if I don't necessarily share my difficulties, the structured interactions within these groups help me find psychological and emotional health. For example, attending scheduled meetings makes me feel connected and motivated.” (Participant 6, female).*

In addition to this sense of motivation, others emphasised how regular and intentional dialogue within organisational settings creates opportunities for self-reflection and personal growth:

*“Just having someone regularly ask me, ‘What do you think about your life? What do you find difficult?’ is helpful in itself. As I answer, I find myself gradually coming to my own conclusions. The complicated thoughts in my mind become organised. Being able to do this through weekly gatherings is very meaningful.” (Participant 11, male).*

### 3.4 Community level: Importance of residential environments to the health and life of middle-aged individuals

The community level included two subcategories: “discomfort from societal expectations” and “seeking safe residential environments for social engagement.” Participants described feeling pressured by societal expectations that middle-aged adults should achieve milestones, such as marriage and parenthood, and experienced emotional distress when these expectations were unmet. They also emphasised the importance of living in safe, socially interactive neighbourhoods to enhance their emotional well-being. Women particularly stressed the need for safety, noting that well-equipped neighbourhoods with cultural facilities, supermarkets, and accessible transportation reduced feelings of loneliness. Examples include:

Subcategory 1: Discomfort from a general perception of lacking achievement in personal development

*“These days, there's a growing societal atmosphere that suggests we shouldn't be asking questions like 'Did you get married? Why not?' But for me, it's like they automatically assume I'm a mom and say things like 'Here, take this for your child.' So, since I don't have a child, it's like saying, 'I'm sorry for not being married.' It feels even worse like it's touching on a sore spot.” (Participant 4, female).*

Beyond these everyday encounters, others described how marital status influenced perceptions of competence and maturity within professional settings, reinforcing subtle forms of stigma:

*“At work, people sometimes say that because I’m unmarried, I look younger… However, at times, especially in meetings where senior executives were present, not being married itself seemed to imply that my social status was somewhat lower or that I was less mature. In those situations, I could sense prejudice and even feelings of stigma.” (Participant 3, male).*

Subcategory 2: Pursuit of a safe residential environment that allows for social interaction

*“In Seoul, there's a lot of infrastructure, but coming to the provinces, there's none... Even in the provinces, if there are well-equipped areas with infrastructure, you can live a much better life. But living in areas that are a bit more remote, as someone who lives alone, feels a bit lonelier and not so good.” (Participant 5, female).*

In addition to concerns about infrastructure and accessibility, participants emphasised the importance of safety and neighbourhood conditions in shaping their residential choices:

*“I think it is important to live in a safe place. Areas with high crime rates or dense one-room housing complexes may be unavoidable when one is young and financially constrained, but once I gain some economic stability, they are places I would prefer to avoid.” (Participant 6, female).*

### 3.5 Public policy level:healthcare utilisation and health management improvement through social safety nets

The public policy level included two subcategories: “life outside the social safety net” and “barriers to healthcare access and the need for improved health literacy.” This theme underscored the lack of policy attention for middle-aged adults living alone. Participants felt overlooked, as most policies target youth, older adults, or families, with the frequent assumption that middle age is a stable life stage. They expressed frustration with this exclusion and highlighted difficulties accessing healthcare without a caretaker, calling for policies that better support independent, healthy living. Examples include:

Subcategory 1: Life outside the social safety net

*“We are outside a safety net. In our 20 s and 30 s, we thought that, by the time we were in our 40 s or 50 s, we would have money and live without worries, but now that we’re in our 40 s and 50 s, it’s nothing like that.” (Participant 9, female).*

Beyond these unmet expectations, participants also expressed frustration with institutional policies that appeared to exclude them, reinforcing their perception of being left unprotected:

*“I feel very dissatisfied. People living alone pay a huge amount in taxes… but the house I live in now is a jeonse contract (a South Korean housing lease system in which tenants pay a large deposit instead of monthly rent). But when I try to buy my own home, all the benefits go to families with multiple children, older adults, newlyweds, or young people. None of the policies apply to me.” (Participant 10, female).*

Subcategory 2: Barriers to single-person household healthcare access and policies needed to improve health literacy

*“I had to be hospitalised urgently due to anaemia, and they told me I needed to have family with me. But I didn’t want that. I consider myself independent, and I thought, if necessary, I would just hire a caregiver... But they insisted I call my family.” (Participant 4, female).*

In addition to institutional barriers that assume family support, participants underscored the lack of accessible resources to support informed health-related decisions in daily life:

*“When I buy food or eat something simple, I don’t really know how much of the essential nutrients I am actually getting. I wish there were services that made this information more easily accessible. Even if there are apps or websites, if they were more user-friendly and naturally integrated into daily life, I think more people could maintain healthier lifestyles.” (Participant 3, male).*

## 4 Discussion

This study used the BPS framework to examine the health and social support needs of middle-aged adults living alone. The central theme was “Overcoming the loneliness of old age through social interaction.” Participants faced aging with limited preparation, shaped by their autonomy and unstructured lifestyles. Unlike older adults who often actively manage their health and combat loneliness (Enjezab et al., 2012), these individuals remained passive and avoidant despite their anxiety about aging. Since midlife health strongly influences successful aging (Rodrigues et al., 2023), tailored interventions are required to address the distinct challenges of this population. This study extends prior research on older adults by focusing specifically on their unique vulnerabilities during midlife.

The first intrapersonal-level theme was autonomy and avoidance in aging. Participants described changes related to aging, a preference for autonomy and ease, and a desire to avoid loneliness in later life. Similar findings were reported in a qualitative study conducted in a large city in Norway (Charpentier & Kirouac, 2022), in which participants valued freedom from caregiving and greater self-control. However, unlike older adults, who often adopt health routines to manage aging, participants in this study expressed concern about future vulnerabilities, especially a lack of caretakers, but remained passive in health management. As noted in prior research, middle-aged adults living alone tend to favour irregular, comfort-driven lifestyles over healthy routines (Charpentier & Kirouac, 2022). However, healthy behaviours are linked to higher life satisfaction (Yoon et al., 2023) and are critical for successful aging (Golchin et al., 2022; Soulières & Charpentier, 2022).

In terms of policy support for improving the lifestyles of middle-aged adults living alone, the participants themselves suggested the need for more accessible tools, such as mobile applications or online platforms that provide user-friendly information on nutrition and health routines. Extending this idea, emerging artificial intelligence (AI) technologies could further enhance accessibility and personalisation by providing tailored health guidance, interactive feedback, and continuous support, thereby addressing both health literacy gaps and the absence of family caregivers. At the same time, the use of such technologies raises important considerations regarding privacy, information reliability, and equitable use, underscoring the need for careful implementation and appropriate ethical oversight (WHO, 2025).

Early aging, marked by emotional detachment and anxiety about the future (Zhang et al., 2024), offers an opportunity to promote healthier habits. Key barriers include lethargy, avoidance, and a comfort-oriented mindset. Autonomy also shapes how individuals experience solitude (Nikitin et al., 2022). Although participants often chose solitude and found it comforting, this was partly because of discomfort in social interactions. To enhance quality of life, it is important to reduce interpersonal challenges, address the downsides of solitary living, and confront social stigma.

Participants acknowledged that having a companion or engaging in community activities supported their health. This aligns with findings from a study conducted in Tehran, Iran, which identified cohabitation as beneficial for healthy aging (Solhi et al., 2022b). Social support plays a crucial role in navigating midlife challenges; however, middle-aged adults living alone often hesitate to actively build relationships. Maintaining social engagement during midlife positively influences later health and life satisfaction (Golchin et al., 2022), suggesting that promoting active social participation may be vital for improving well-being in this group (Sirén et al., 2023). For example, municipalities could develop community-based programs, such as arts, sports, travel, reading, or faith-based activities that foster achievement, vitality, and natural participation, thereby encouraging interpersonal engagement during midlife. In addition, integrating AI-based tools into such initiatives—for instance, AI-powered platforms that recommend personalised community activities or provide digital companionship—may offer novel ways to engage socially isolated individuals and reduce barriers to participation.

Shorey and Chan (2021) found that older Asian adults often use religious practices and peer engagement to cope with loneliness and actively join community programs to reduce isolation. By contrast, this study revealed that middle-aged adults living alone engage with communities more for self-perception, vitality, and self-reflection, thus reflecting developmental tasks specific to midlife. Despite this, participants showed a tendency toward voluntary isolation and passivity, hoping for relationships to form naturally rather than actively pursuing them. These findings highlight the need for interventions that encourage proactive social engagement and support personal growth in this population.

In the sociocultural context of this study, participants described negative societal perceptions of middle-aged adults living alone. In many Asian cultures, including South Korea, family is highly valued, and living alone is often stigmatised (Ngoo et al., 2015). Marital status remains a key factor in perceived life satisfaction among middle-aged individuals (Ngoo et al., 2015). However, due to growing economic and social pressures, attitudes toward marriage in South Korea have been shifting, with more people viewing it as optional (Hong, 2020). In parallel, the 4B movement refers to *bihon* (no marriage), *bichulsan* (no childbirth), *biyeonae* (no dating), and *bisekseu* (no sex). It originated in South Korea and has been discussed as a sociocultural discourse reflecting resistance to gendered social expectations, particularly in online spaces (Schepens, 2025). This study reflects an increasing acceptance of independent lifestyles, although lingering societal stigma continues to cause discomfort and alienation, contributing to social isolation and potential mental health challenges (Kotozaki & Levy-Storms, 2014).

This study underscores that current social policies largely focus on youth, families, and older adults, thus overlooking middle-aged adults living alone. The participants stressed the importance of safe, socially engaging residential environments for their well-being. These findings are consistent with a study of Seoul residents in South Korea (Lee & Eom, 2018), especially concerning women’s safety. As living environments influence values, daily activities, and life satisfaction (Foye, 2017), limited housing options may restrict opportunities for social interaction. Understanding the role of residential context is crucial for promoting well-being in this population. Rising housing costs in South Korea, along with policies favoring youth, newlyweds, and older adults, have affected marginalised middle-aged adults living alone. The theme of “life outside the social safety net” for this study highlights critical policy gaps. The findings indicate an urgent need to address housing and healthcare policy gaps to ensure equitable access without dependence on caretakers.

The findings at the intrapersonal level reveal how loneliness and anxiety, compounded by a lack of caretakers, create additional barriers to essential medical services. Participants noted that hospital policies requiring caretaker consent for admission posed significant barriers, particularly for older adults living alone who face frequent hospital visits. Given the increasing number of individuals living alone in South Korea (Yeung & Cheung, 2015), revising hospital policies regarding caretaker requirements is imperative for enhancing accessibility to medical services and mitigating health disparities. Policies could expand integrated nursing care services with priority access and reduce costs for middle-aged adults living alone, ensuring safe inpatient care without family support. Additionally, AI technologies, such as home nursing programs, monitoring systems, and chatbots, could provide continuous post-discharge care without reliance on caretakers.

### 4.1 Limitations

Although this study offers meaningful insights, it has several limitations. First, it explored current health needs, but did not examine the long-term experience of living alone in middle age. Longitudinal qualitative studies are required to address this gap. Second, as the study focused on the South Korean context, cultural and socioeconomic differences may limit its generalisability. Third, the small, nonrepresentative sample lacked diversity in terms of health status, sex, and socioeconomic background. Although some demographic data (e.g. income, education, and chronic disease) were collected, other relevant factors and specific living conditions were not fully explored. Future research should consider a broader range of variables to better inform targeted policy developments.

## 5 Conclusion

This qualitative study investigated the biopsychosocial health needs of middle-aged adults living alone by exploring their health experiences. The findings indicate that they face autonomy and avoidance in aging, largely due to the absence of a caretaker. Participants emphasised the importance of social connections, community involvement, and a safe, socially engaging home environment. Many often perceive themselves as outside the traditional social protection framework, highlighting the need for tailored social support policies. Such policies should focus on reducing fears of aging alone, increasing social participation, and ensuring a safe and supportive residential environment. This study is significant because it identifies the specific health needs of middle-aged adults living alone and provides evidence for developing customised policies to meet these needs.

## Data Availability

Data cannot be shared openly but are available upon request from the authors.
